# Disturbance structures canopy and understory productivity along an environmental gradient

**DOI:** 10.1111/ele.13849

**Published:** 2021-08-02

**Authors:** Max C. N. Castorani, Shannon L. Harrer, Robert J. Miller, Daniel C. Reed

**Affiliations:** ^1^ Department of Environmental Sciences University of Virginia Charlottesville VA USA; ^2^ Marine Science Institute University of California Santa Barbara CA USA

**Keywords:** canopy, competition, disturbance, environmental gradient, habitat quality, kelp forest, long‐term experiment, net primary productivity, shading, understory

## Abstract

Disturbances often disproportionately impact different vegetation layers in forests and other vertically stratified ecosystems, shaping community structure and ecosystem function. However, disturbance‐driven changes may be mediated by environmental conditions that affect habitat quality and species interactions. In a decade‐long field experiment, we tested how kelp forest net primary productivity (NPP) responds to repeated canopy loss along a gradient in grazing and substrate suitability. We discovered that habitat quality can mediate the effects of intensified disturbance on canopy and understory NPP. Experimental annual and quarterly disturbances suppressed total macroalgal NPP, but effects were strongest in high‐quality habitats that supported dense kelp canopies that were removed by disturbance. Understory macroalgae partly compensated for canopy NPP losses and this effect magnified with increasing habitat quality. Disturbance‐driven increases in understory NPP were still rising after 5–10 years of disturbance, demonstrating the value of long‐term experimentation for understanding ecosystem responses to changing disturbance regimes.

## Graphical abstract

In a decade‐long field experiment, we show that habitat quality can mediate the effects of intensified disturbance on canopy and understory net primary productivity (NPP) in kelp forests. Experimental annual and quarterly disturbances suppressed total macroalgal NPP; however, understory macroalgae partly compensated for canopy NPP losses and this effect magnified with increasing habitat quality. Disturbance‐driven increases in understory NPP were still rising after 5–10 years of disturbance, demonstrating the value of long‐term experimentation for understanding ecosystem responses to changing disturbance regimes.

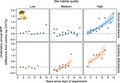

## INTRODUCTION

Net primary productivity (NPP)—the rate at which autotrophs produce new organic material—is a fundamental measure of ecosystem function that determines organic carbon accumulation and availability to consumers (Cebrian, 1999; Cebrian & Lartigue, 2004; Pace & Lovett, 2013). Despite decades of study (Kira et al., 1969; Rosenzweig, 1968; Whittaker, 1961), understanding the patterns and controls of NPP has been hindered by the complexities of measuring and manipulating productivity across vertically stratified vegetation layers that characterise many terrestrial and aquatic ecosystems (Clark et al., 2001; Gower et al., 2001; Miller et al., 2011). Addressing this challenge is important because canopy and understory vegetation differ in their: (1) rate, form and fate of primary production (Frost et al., 1997; Kelty, 1989; Misson et al., 2007); (2) ability to compete for limited resources (Bloor & Grubb, 2003; Kitajima, 1994; Poorter et al., 2003) and (3) response to various forms of disturbance (Dale et al., 2001; Hart & Chen, 2006; Walker et al., 2010). Resolving how and under what circumstances disturbance structures NPP and its allocation among vegetation layers is urgent because humans are altering disturbance regimes and environmental conditions in many ecosystems (Dale et al., 2001; Gaiser et al., 2020; Harris et al., 2018).

Disturbances can shift the balance of NPP among vegetation layers directly by disproportionately removing vegetation from certain layers, and indirectly by changing resource availability and competitive interactions (Alaback, 1982; Hart & Chen, 2006; Miller et al., 2011). Although canopy productivity often dominates ecosystem productivity (Gower et al., 2001; Misson et al., 2007; Nilsson & Wardle, 2005; Tait & Schiel, 2018; Wiesner et al., 2019), intermittent or sustained disturbances to upper vegetation layers can promote understory NPP that rivals that of the canopy, with cascading community and ecosystem effects (Alaback, 1982; Hart & Chen, 2006; Lloyd et al., 2008; Miller et al., 2011; Nilsson & Wardle, 2005). For example, storms periodically reduce forest aboveground NPP by destroying trees, but create canopy gaps that increase light and boost herbaceous understory NPP, thereby altering decomposition (Kennard et al., 2020; Muscolo et al., 2014; Royo & Carson, 2006). Conversely, climate warming can alter competition to change low, grassy habitats into tall, closed‐canopy wooded ecosystems (Cavanaugh et al., 2014; Huang et al., 2018; Myers‐Smith et al., 2011), changing the amount, form and fate of NPP (Knapp et al., 2008; Mekonnen et al., 2018).

However, the impact of disturbance on NPP may change if biotic or abiotic factors associated with habitat quality (i.e. conditions that promote species persistence; Hall et al., 1997) alter the amount of vegetation susceptible to disturbance (Roberts, 2004; Royo & Carson, 2006). For example, gradients in grazing, precipitation and soils can strengthen or diminish the effects of fire on grassland productivity by changing the abundance and composition of vegetation before and after disturbance (Briggs et al., 2002; Collins & Calabrese, 2012; Fuhlendorf & Smeins, 1997; Fynn et al., 2005; Oesterheld et al., 1999). Environmental conditions can also alter the effects of disturbance on NPP by constraining the potential for understory vegetation to compensate for losses of canopy productivity (Frost et al., 1997; Halpern & Lutz, 2013; Hughes et al., 2006; Royo & Carson, 2006). Nevertheless, knowledge of the degree to which disturbance effects on NPP are mediated by habitat quality is largely limited to systems with relatively simple vegetation structure, such as grasslands, that respond rapidly and are amenable to experimentation (Collins & Calabrese, 2012; Haddad et al., 2002; House et al., 2003; Oesterheld et al., 1999; Seabloom et al., 2020). Ecosystems with large, complex canopies, such as forests, are logistically difficult to manipulate at ecologically meaningful spatial scales and can require decades to centuries of study to evaluate the response of ecosystem NPP to disturbance. Consequently, the roles of disturbance and environmental conditions in altering forest NPP have usually been studied indirectly using chronosequences and associated space‐for‐time substitutions that allow limited inference (Johnson & Miyanishi, 2008; Walker et al., 2010). Moreover, across a variety of ecosystems, most investigations have not achieved the long durations needed to determine how canopy and understory NPP change over the multiple cycles of disturbance and recovery that occur when disturbance regimes shift in frequency or severity (Donohue et al., 2016; Haddad et al., 2002; Knapp et al., 2012). Hence, the potential for understory vegetation to compensate for sustained losses of canopy productivity under intensified disturbance regimes is largely unknown, particularly for systems with large, complex canopies (House et al., 2003; Reich et al., 2001; Wiesner et al., 2019).

Several of the challenges to understanding the effects of disturbance on canopy and understory NPP and how they vary along environmental gradients can be overcome through the study of marine forests formed by canopy‐forming kelps and associated understory macroalgae. Such kelp forests consist of multiple vegetation layers with complex vertical structure and canopy heights comparable to most terrestrial forests (5–30 m; Lefsky, 2010; Schiel & Foster, 2015). These macroalgal assemblages can exhibit very high NPP (>4 kg C/m^2^/y; this study) and are comprised of relatively short‐lived species that vary in their susceptibility to intermittent (e.g. storm‐driven waves) and sustained (e.g. ocean warming) disturbances (Dayton et al., 1992; Ebeling et al., 1985; Harris et al., 2018). The biomass and productivity of different vegetation layers are further influenced by biotic and abiotic factors that affect habitat quality (e.g. herbivory and substrate suitability; Lamy et al., 2018; Miller et al., 2018; Schiel & Foster, 2015). Habitat quality for understory and canopy‐forming macroalgae has been degraded in many regions as a result of changes in sediment dynamics and herbivore density (Airoldi, 2003; Filbee‐Dexter & Scheibling, 2014), and climate change is predicted to bring about warmer seas and more destructive storms that negatively affect canopy‐forming macroalgae (Ummenhofer & Meehl, 2017).

Here, we use giant kelp forests to determine how canopy disturbance and habitat quality interact to structure NPP dynamics across canopy and understory vegetation layers. We carried out a uniquely long‐term (5–10 years) field experiment in which we tracked the response of macroalgal NPP to annual and quarterly canopy disturbances in kelp forests occurring along a gradient in habitat quality arising from spatial variation in herbivore density and substrate suitability. Our results suggest that increasing intermittent or sustained losses of canopy vegetation caused by climate change and intensifying disturbance regimes will depress ecosystem NPP, and that the ability of understory vegetation to compensate will be strongly mediated by environmental gradients.

## MATERIAL AND METHODS

### Study system

The giant kelp *Macrocystis pyrifera* is the largest and most widely distributed kelp species, forming extensive, highly productive forests on shallow temperate reefs worldwide (Graham et al., 2007; Schiel & Foster, 2015). As a foundation species, giant kelp influences coastal ecosystem structure and function (Lamy et al., 2020; Miller et al., 2018) by modifying the surrounding environment, especially through canopy shading that reduces light at the seafloor (Castorani et al., 2018; Clark et al., 2004; Dean, 1985; Desmond et al., 2015; Watanabe et al., 1992). Relative to understory macroalgae, giant kelp suffers greater loss from wave disturbance (Dayton et al., 1992; Ebeling et al., 1985) because it spans the entire water column and experiences strong drag (Seymour et al., 1989). Giant kelp and understory macroalgae also respond differently to prolonged disturbances like warming (Harris et al., 2018). Because marine macroalgal growth is often light limited (Harrer et al., 2013; Miller et al., 2011; Tait & Schiel, 2018), giant kelp canopy loss can increase the abundance and diversity of understory macroalgae (Castorani et al., 2018; Schiel & Foster, 2015).

### Study sites

We worked at five reefs studied by the Santa Barbara Coastal Long Term Ecological Research Project (SBC LTER) near Santa Barbara, California, USA: Mohawk (119.73 °W, 34.39 °N), Isla Vista (119.86 °W, 34.40 °N), Arroyo Quemado (120.12 °W, 34.47 °N), Naples (119.95 °W, 34.42 °N) and Carpinteria (119.54 °W, 34.39 °N). Sites were separated by 9–54 km, were 6–9 m deep (mean lower low water) and represented a range of physical and biological characteristics that influence subtidal macroalgal assemblages in the region. Measurements and experiments were carried out for 10 years at Mohawk, Arroyo Quemado and Carpinteria (2008–2018); 9 years at Naples (2008–2017) and 5 years at Isla Vista (2012–2017).

### Macroalgal habitat quality

Sea urchin grazing and substrate type are among the most important local determinants of macroalgal abundance and diversity at our sites (Miller et al., 2018) and on temperate reefs in general (Dayton, 1985; Witman & Dayton, 2001). In particular, increases in sand cover on reefs create habitat that is largely unsuitable for the attachment and survival of giant kelp and most understory macroalgae (Airoldi, 2003; Miller et al., 2018; Reed et al., 2008). Sand cover on reefs in our system is highly dynamic as a result of spatiotemporal variation in reef topography and sediment transport processes. As per Hall et al., (1997), we define habitat quality as ‘the ability of the environment to provide conditions appropriate for individual and population persistence … [represented by] a continuous variable, ranging from low to medium to high’. Hence, we modelled total macroalgal biomass as a function of sea urchin density and sand cover, and used model predictions to characterise a continuous index of macroalgal habitat quality at each site (see *Statistical analyses*).

At each site, divers measured sea urchin density (*Strongylocentrotus purpuratus* and *Mesocentrotus franciscanus*), proportional sand cover and macroalgal biomass (giant kelp and 56 understory taxa) within a permanent 40 × 2 m transect once per season as defined by the typical solar solstice and equinox dates: winter (Dec. 21–Mar. 19), spring (Mar. 20–June 20), summer (June 21–Sep. 21) and autumn (Sep. 22–Dec. 20). Different non‐destructive methods were used to quantify macroalgal abundance depending on species size and morphology (see Appendix S1). Divers counted the number and measured the size of all giant kelp and larger understory kelps within each transect. The abundances of smaller kelps and sea urchins were subsampled in six permanent 1 m^2^ quadrats spaced uniformly along each transect. Substrate and the abundances of small or clonal macroalgae that are difficult to distinguish as individuals were measured as per cent cover using a grid of 80 points spaced uniformly within a 1‐m‐wide band spanning each transect. We converted size‐specific abundance and per cent cover measurements to dry mass using taxon‐specific relationships (Nelson et al., 2021; Reed & Miller, 2021a), and giant kelp frond density to dry mass using allometric relationships developed for each month of the year (Rassweiler et al., 2018; see Appendix S1).

### Disturbance experiment

To assess the effects of canopy disturbance on kelp forest productivity, we repeatedly removed giant kelp by hand annually or quarterly from permanent plots at each site (one plot per treatment per site; see Appendix S2 for amount of giant kelp biomass periodically removed). Annual disturbance plots were 1500–2000 m^2^ (50 × 30–40 m) and quarterly disturbance plots were 500 m^2^ (50 × 10 m). An adjacent unmanipulated 2000 m^2^ (50 × 40 m) plot served as a control at each site.

The annual disturbance treatment was carried out once per year in winter (January to February) to mimic large, storm‐driven winter waves that cause giant kelp mortality (Reed et al., 2008, 2011). Such annually recurrent disturbances to giant kelp are typical throughout its range in regions exposed to large seasonal swells (Graham et al., 2007; Schiel & Foster, 2015). The quarterly disturbance treatment was performed in fixed plots once or twice per season (4–8 times per year) to mimic near continual loss of giant kelp, which can result from prolonged ocean warming and other press disturbances (Butler et al., 2020; Cavanaugh et al., 2019). Climate change may bring about warmer, stormier seas that would increase both intermittent and sustained kelp canopy losses (Ummenhofer & Meehl, 2017).

Control and annual disturbance plots were followed for 10 years (2008–2018) at Mohawk, Arroyo Quemado and Carpinteria; 9 years (2008–2017) at Naples and 5 years at Isla Vista (2012–2017). Quarterly disturbance plots were followed for 7 years (2010–2017) at Mohawk, Arroyo Quemado and Carpinteria and 6 years (2010–2016) at Naples. The quarterly disturbance treatment was not established at Isla Vista.

### Estimating net primary productivity

In each plot, we estimated daily NPP of juvenile giant kelp (<1 m tall) and understory macroalgae by combining *in situ* measurements of hourly irradiance and taxon‐specific biomass with taxon‐specific relationships between irradiance and photosynthesis, accounting for respiration (details in Appendix S1 and Harrer et al., 2021; validation in Miller et al., 2012). We measured light 30 cm above the seafloor in each plot every 1–2 minutes using a photosynthetically active radiation (PAR) logger sensor mounted to a stake (see Appendix S1). We non‐destructively measured macroalgal biomass within permanent 40 × 2 m transects centred within each plot (one transect per plot) as described previously and estimated the daily standing biomass of each taxon using linear interpolations between sampling dates. We calculated taxon‐specific NPP as the sum of gross production and respiration over all hours of daylight, and respiration over all hours of darkness for each day of the year as per Miller et al., (2012):

$${\mathrm{NPP}}_{i}=\sum_{h}P_{\max}\cdot \tanh\left(\frac{\alpha_{i}E_{h}}{P_{\max}}\right)\cdot b_{i}‐\sum_{h}R\cdot b_{i},$$

where *P*_max_ is net photosynthesis at saturating irradiance (mg C h^−1^ [g dry mass]^−1^), *α_i_* is net photosynthesis at non‐saturating irradiance (mg C h^−1^ [g dry mass]^−1^ [µmol m^−2^ s^−1^]^−1^), *E_h_* is mean seafloor irradiance (µmol m^−2^ s^−1^) over the course of an hour (*h*), *R* is respiration in the dark (mg C h^−1^ [g dry mass]^−1^) and *b_i_* is the daily estimate of standing dry biomass (g m^−2^) of an individual taxon *i*.

We estimated NPP of adult giant kelp (>1 m tall) by multiplying interpolated daily biomass by the month‐specific slope between biomass and mean daily NPP (Rassweiler et al., 2018). We summed taxon‐specific estimates of daily NPP from early spring of one calendar year (after initiating experiments in midwinter) through late winter of the following calendar year to produce annual NPP values for each year following the start of the experiment.

### Statistical analyses

We compared sea urchin density among sites using a generalised least squares (GLS) model with first‐order autoregressive correlation structure [AR(1)] while controlling for the effect of year (Pinheiro & Bates, 2000; Zuur et al., 2009). We compared proportional sand cover among sites using a zero‐inflated (ZI) beta generalised linear model (GLM) with a logit link function, and total macroalgal biomass using an AR(1) GLS model. We quantified the combined effects of sea urchin density and sand cover on macroalgal biomass using a Gamma GLM with a log link function. We then made predictions from this model using measurements of sea urchin density and sand cover to create a continuous macroalgal habitat quality index (rescaled to a maximum of one), and used a linear model to compare values of this variable among sites.

To understand how natural fluctuations in canopy biomass affect bottom light to influence understory NPP, we examined average daily irradiance in control plots as a function of giant kelp biomass over a 15‐day window centred around each sampling event (i.e. the day of sampling ±1 week). We used an AR(1) Gamma GLM to relate bottom PAR to giant kelp biomass, season and their interaction while controlling for effects of site and year.

**FIGURE 1:**
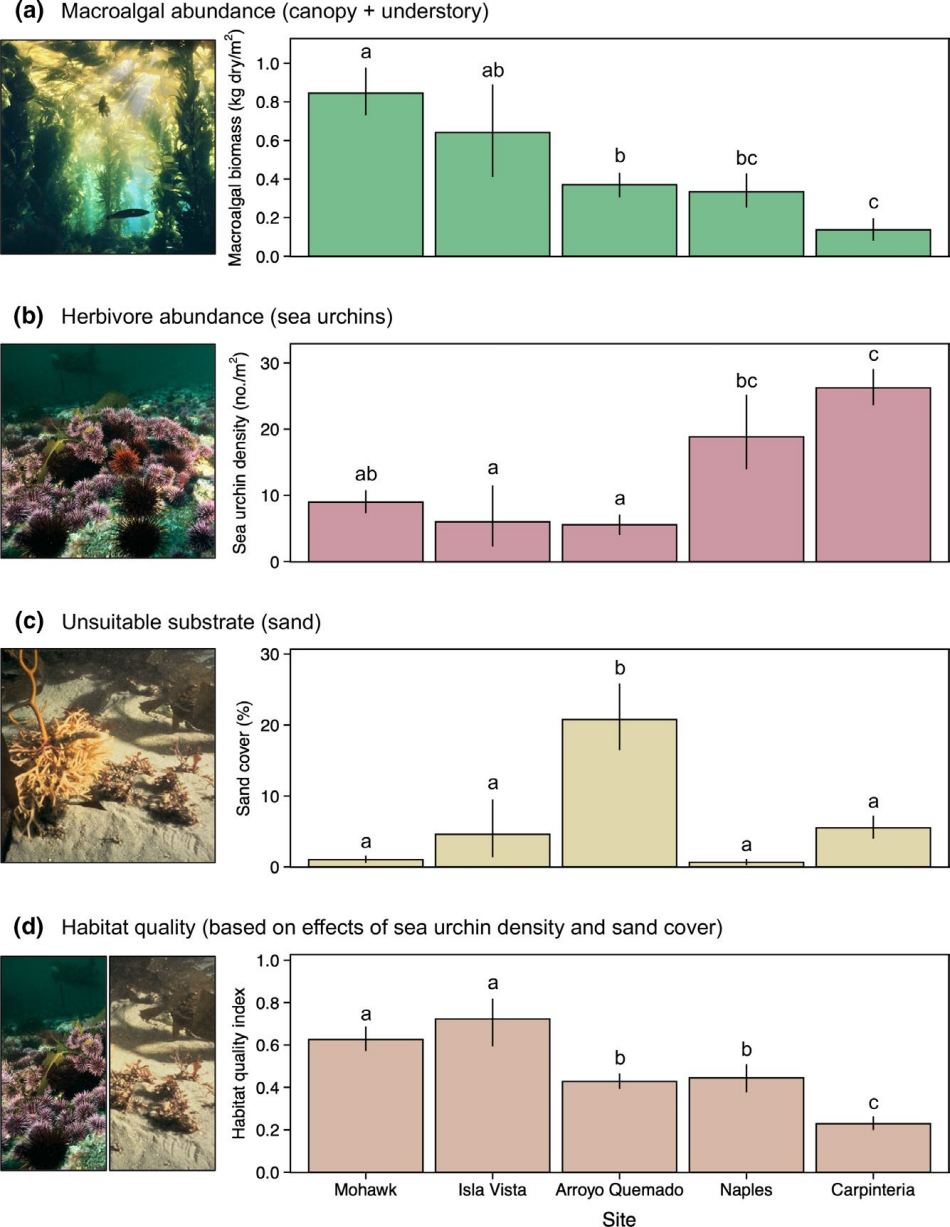
(a) A gradient in macroalgal biomass was structured by among‐site variation in (b) the density of herbivorous sea urchins and (c) the cover of sand that is largely unsuitable for the attachment and survival of giant kelp and most understory macroalgae. Habitat quality, based on the effects of sea urchins and sand cover, is shown in panel (d) (rescaled to a maximum of one; see Appendix S3). Data represent 10 years (2008–2018) of seasonal observations from unmanipulated control plots at five kelp forest sites near Santa Barbara, California, USA. Bars and error bars show mean ± bootstrap 95% confidence intervals for seasonal measurements. Letters indicate pairwise differences between sites (*p* < 0.05). Photo credit: Ron H. McPeak

To test our central hypothesis that the effects of canopy disturbance on NPP dynamics are mediated by habitat quality, we analysed changes in annual NPP over the course of the experiment as a function of disturbance regime and macroalgal habitat quality. To quantify the variation in NPP attributable to annual and quarterly disturbances, we calculated the difference in annual NPP between paired control and disturbance plots at each site for giant kelp, understory macroalgae and total macroalgae (i.e. giant kelp and understory macroalgae combined). Analysis of macroalgal habitat quality indicated that sites clustered into three levels of habitat quality (see *Results*; Figure 1d): low (Carpinteria), medium (Arroyo Quemado and Naples) and high (Mohawk and Isla Vista). Thus, we analysed how the difference in annual NPP between paired control and disturbance plots varied as a function of time since the start of the experiment, habitat quality (low, medium and high), disturbance regime (annual and quarterly) and the two‐ and three‐way interactions among these variables. We used a Gamma GLM for understory NPP and GLS models for giant kelp and total macroalgal NPP.

Lastly, we quantified the relationship between the annual mean biomass of giant kelp and annual NPP of the understory and total macroalgal community based on data from all treatments combined using an AR(1) Gamma GLM and an AR(1) GLS model, respectively, with covariates to control for the effects of sea urchin density, sand cover and year.

We analysed models in R 4.0.3 (R Core Team, 2020) using *glmmTMB* 1.0.2.1 (Magnusson et al. 2020). We assessed the significance of model terms using Wald chi‐squared and *F* tests (Zuur et al., 2009). We used *post hoc t* tests to estimate effects and pairwise differences within and among factor levels. We checked for homogeneity of variance by plotting normalised model residuals against model predictions and individual predictors, and specified heterogeneous covariance structures where needed (Pinheiro & Bates, 2000). We ensured normality of residuals using histograms and quantile–quantile plots, and square‐root transformed response variables where necessary. We estimated effect sizes based on model predictions that control for the influence of covariates, autocorrelation and heterogeneity of variance using *emmeans* 1.4.5 (Lenth, 2020). Our analyses consisted of several statistical tests, increasing the likelihood of false positives; thus, we adjusted all *p* values to control the false discovery rate (Benjamini & Hochberg, 1995).

## RESULTS

### Gradient in habitat quality across sites

Macroalgal biomass in control plots varied systematically among sites in association with gradients in sea urchin density and sand cover (Figure 1). One‐third of observed variation (*R* ^2^ = 0.33) in total macroalgal biomass (Figure 1a) was explained by negative associations with sea urchin density ($\chi_{1,172}^{2}$ = 140.3; *p* < 0.001) and sand cover ($\chi_{1,172}^{2}$ = 22.1; *p* < 0.001). In particular, macroalgal biomass was highly variable when sea urchin density was less than ~25 urchins/m^2^ or sand cover was less than ~5–10%, but consistently low at higher values (Appendix S3). Thus, variation among sites in sea urchin density (Figure 1b; $\chi_{4,156}^{2}$ = 41.6; *p* < 0.001) and sand cover (Figure 1c; $\chi_{4,169}^{2}$ = 62.8; *p* < 0.001) resulted in differences in macroalgal habitat quality (Figure 1d; *F*_4,171_ = 36.7; *p* < 0.001). Habitat quality was highest at Mohawk and Isla Vista (*t*_1,171_ ≥ 4.4; *p* < 0.001), intermediate at Arroyo Quemado and Naples (*t*_1,171_ ≥ 4.4; *p* < 0.001) and lowest at Carpinteria (*t*_1,171_ ≥ 5.0; *p* < 0.001); macroalgal biomass declined in the same order (Figure 1a).

### Effects of giant kelp on bottom light

**FIGURE 2:**
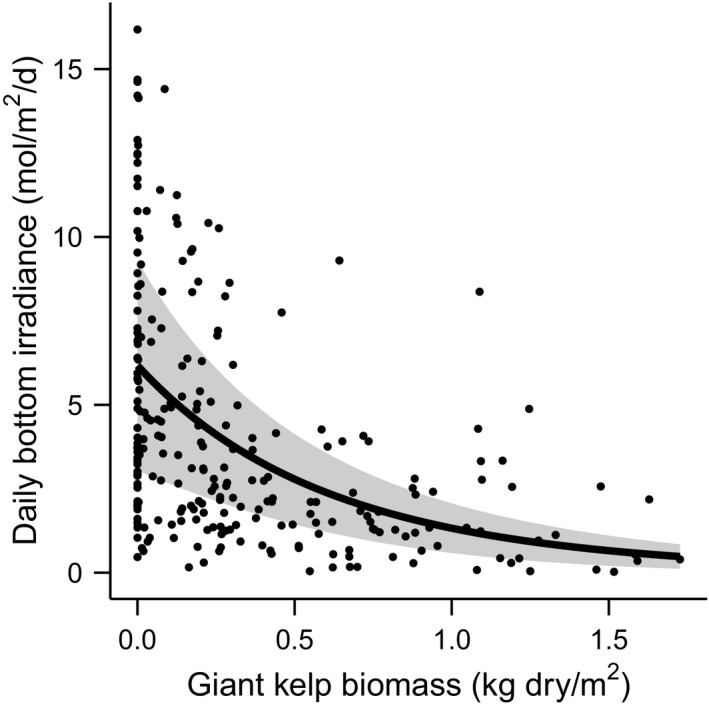
The amount of light reaching the kelp forest seafloor declined exponentially with increasing giant kelp biomass. Data represent seasonal measurements in control plots at all five sites averaged over 15‐day windows centred on point estimates of giant kelp biomass. Line and shading show estimated relationship and 95% confidence interval respectively. See Appendix S5 for trends by season

Light reaching the seafloor declined exponentially with increasing giant kelp biomass in control plots (Figure 2; Appendix S4; $\chi_{1,225}^{2}$ = 76.2; *p* < 0.001). The magnitude of this decline varied slightly among seasons (Appendices S4–S5; $\chi_{3,225}^{2}$ = 114; *p* = 0.001), with 87–99% less light at seasonal maximum giant kelp biomass compared to the minimum (no giant kelp). Based on the long‐term mean giant kelp biomass in experimental plots (averaged over all seasons and years), the model predicted that bottom light increased by 35% with annual disturbance (4.6 vs. 3.4 mol/m^2^/d) and 77% with quarterly disturbance (6.0 vs. 3.4 mol/m^2^/d).

### Effects of canopy disturbance and habitat quality on productivity

**FIGURE 3:**
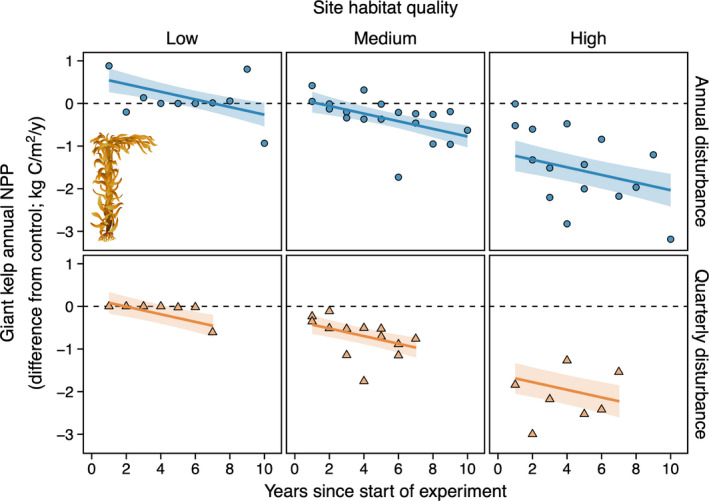
Habitat quality mediated the effect of canopy disturbance on the annual net primary productivity (NPP) dynamics of giant kelp. Annual and quarterly disturbances to canopy‐forming giant kelp decreased giant kelp NPP over time relative to control plots. The rates of these declines were similar irrespective of habitat quality, but overall reductions in giant kelp NPP were greater where habitat quality was higher because there was more giant kelp available for experimental removal. Quarterly disturbances resulted in greater decreases in giant kelp NPP than annual disturbances. Horizontal dashed line indicates equal annual NPP in paired control and disturbance plots. Solid lines and shading as in Figure 2

**FIGURE 4:**
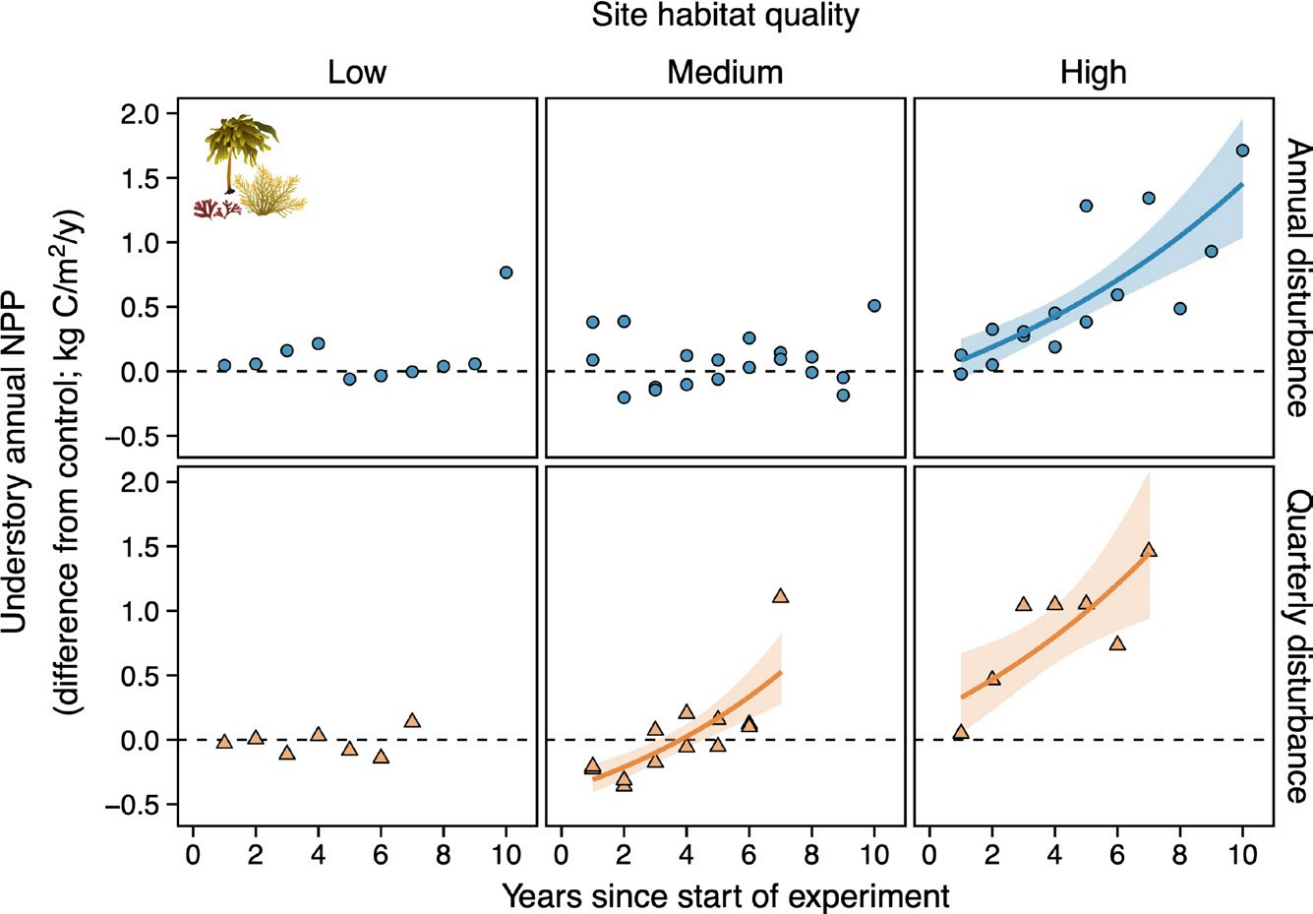
Habitat quality mediated the effect of canopy disturbance on the annual net primary productivity (NPP) dynamics of understory macroalgae. In high‐quality habitat, annual and quarterly disturbances to canopy‐forming giant kelp increased understory NPP over time relative to controls. In medium‐quality habitat, understory NPP increased over time relative to controls with quarterly but not annual disturbance. Disturbance had no effect on understory NPP in low‐quality habitat. Horizontal dashed line, solid lines and shading as in Figure 3

**FIGURE 5:**
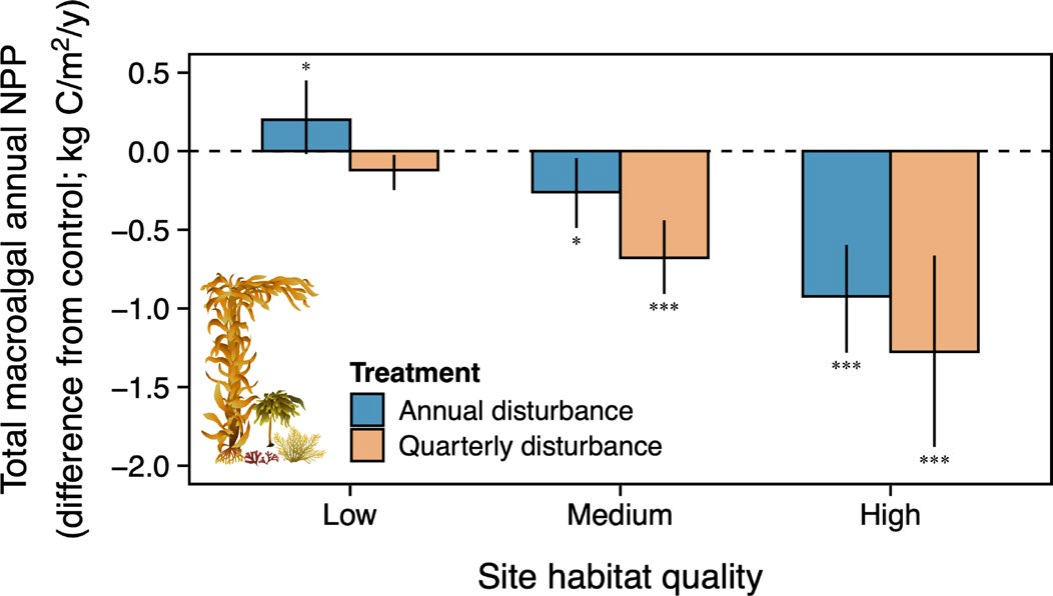
Habitat quality mediated the effects of canopy disturbance on the annual net primary productivity (NPP) of the total macroalgal community (i.e. giant kelp and understory combined). Sites with high habitat quality, and thus, a large amount of giant kelp susceptible to disturbance (Figure 1), suffered large decreases in total NPP relative to controls. Decreasing habitat quality was matched by weaker disturbance‐driven changes in total NPP. Both disturbance regimes reduced total NPP relative to controls, but quarterly disturbances resulted in larger decreases than annual disturbances. Horizontal dashed line as in Figure 3. Bars and error bars show means across all years ± bootstrap 95% confidence intervals. Temporal averages of the data are shown because trends over time were not significant (Appendices S6, S8). Symbols indicate the level of significance in the difference from zero in *post hoc* tests (*p < 0.05; **p < 0.01; ***p < 0.001)

Habitat quality mediated the effects of canopy disturbance on kelp forest NPP (Figures 3, 4, 5; Appendices S6–S7). Annual and quarterly disturbances diminished giant kelp NPP over time relative to controls (Figure 3; main effect of time: $\chi_{1,56}^{2}$ = 19.1; *p* < 0.001). The rates of these declines were similar irrespective of disturbance type or habitat quality (i.e. no difference in slopes in Figure 3; two‐ and three‐way interactions: $\chi_{1‐2,56}^{2}$ ≤ 2.9; *p* ≥ 0.3). However, time‐averaged reductions in giant kelp NPP were greatest in high‐quality habitat, where the biomass of giant kelp and its subsequent loss from experimental removals were highest (compare intercepts in Figure 3; main effect of habitat quality: $\chi_{1,56}^{2}$ = 111.1; *p* < 0.001; *post hoc* test of difference from zero: *t*_1,56_ ≥ 7.8; *p* < 0.001), intermediate in medium‐quality habitat (*t*_1,56_ ≥ 3.0; *p* ≤ 0.007) and no different than control plots in low‐quality habitat (*t*_1,56_ ≤ 1.1; *p* = 0.4). Not surprisingly, quarterly disturbances suppressed giant kelp NPP more than annual disturbances (main effect of treatment: $\chi_{1,56}^{2}$ = 17.9; *p* < 0.001) across all levels of habitat quality (two‐way interaction: $\chi_{1,56}^{2}$ = 2.0; *p* = 0.4).

Habitat quality mediated the effects of canopy disturbance on understory NPP over time (Figure 4; three‐way interaction: $\chi_{1,58}^{2}$ = 13.3; *p* = 0.003). In high‐quality habitat, annual and quarterly disturbances caused annual understory NPP to increase over time relative to controls (*post hoc* test of difference in slope from zero: *t*_1,58_ ≥ 3.1; *p* < 0.005). In medium‐quality habitat, understory NPP increased over time relative to controls with quarterly disturbance (*t*_1,58_ = 5.6; *p* < 0.001) but not annual disturbance (*t*_1,58_ = 0.2; *p* = 0.8). Disturbances had no effect on understory NPP in low‐quality habitat (*t*_1,56_ ≤ 1.5; *p* ≥ 0.2).

Habitat quality also mediated the effects of canopy disturbance on total macroalgal NPP (Figure 5). Sites with high habitat quality suffered large overall decreases in total annual NPP relative to controls and decreasing habitat quality was matched by weaker disturbance‐driven changes in total NPP (main effect of habitat quality: $\chi_{2,56}^{2}$ = 63.4; *p* < 0.001). These declines were roughly twice as large for quarterly disturbances than annual disturbances (main effect of treatment: $\chi_{1,56}^{2}$ = 17.0; *p* < 0.001). However, unlike temporal trends seen in understory NPP and canopy NPP, total macroalgal NPP in disturbance plots did not change over time relative to controls regardless of habitat quality or disturbance regime (main effect and interactions: $\chi_{1‐2,56}^{2}$ ≤ 5.3; *p* ≥ 0.08; Appendix S8).

### Relationship between giant kelp biomass and macroalgal NPP

**FIGURE 6:**
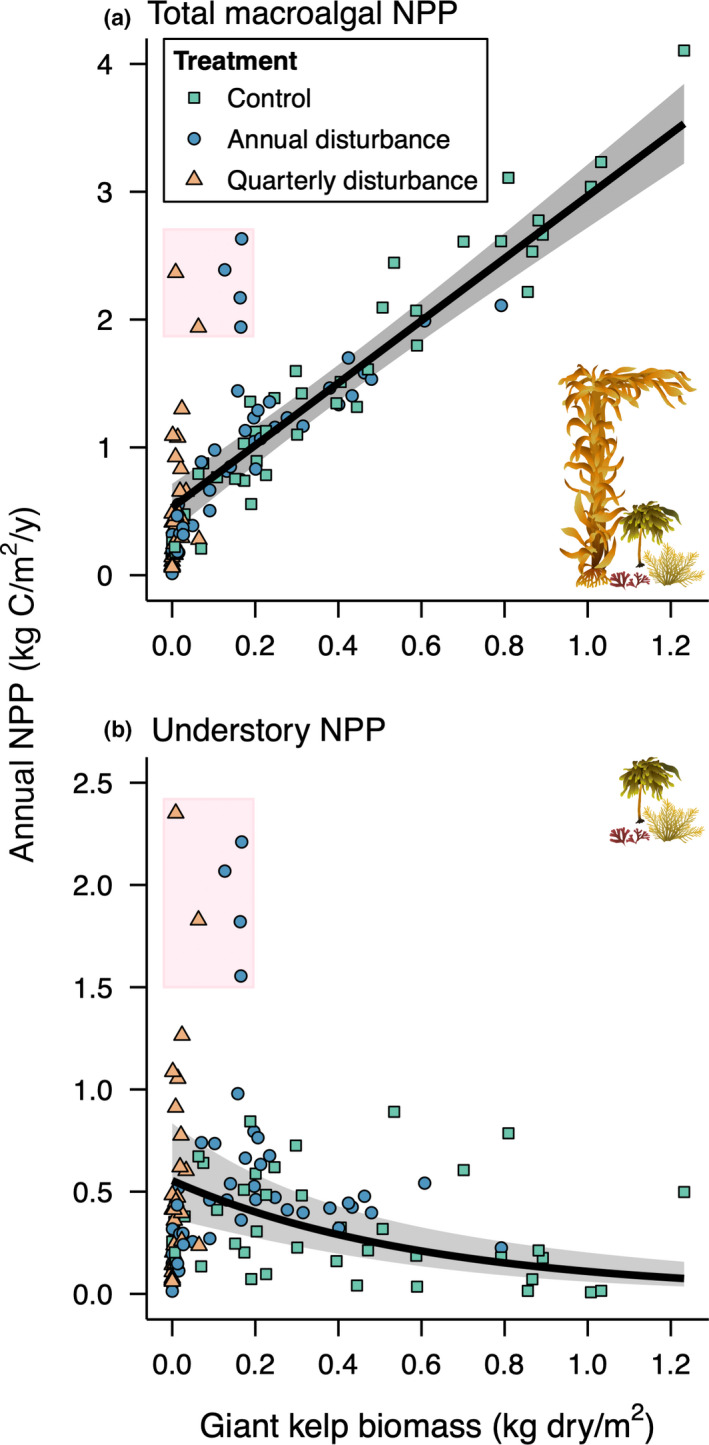
Increasing giant kelp biomass was associated with (a) a linear increase in annual net primary productivity (NPP) by the total macroalgal community and (b) an exponential reduction in annual NPP by understory macroalgae. Values obtained from different treatments are distinguished by different symbols and colours. Shaded boxes highlight observations in annual and quarterly disturbance plots of (a) relatively high total NPP resulting from (b) very high understory NPP. Note differences in the scale of *y*‐axes between panels. Lines and shading as in Figure 2

Because of the high productivity of giant kelp, increasing giant kelp biomass was matched by a linear increase in total macroalgal NPP (Figure 6a; Appendix S9; $\chi_{1,101}^{2}$ = 224.0; *p* < 0.001). On average, total macroalgal NPP was seven times higher when giant kelp was at maximum biomass relative to when it was absent (3.5 vs. 0.5 kg C/m^2^/y). By contrast, increasing giant kelp biomass was associated with an exponential reduction in understory NPP (Figure 6b; Appendix S9; $\chi_{1,107}^{2}$ = 25.2; *p* < 0.001). On average, understory annual NPP was 86% lower when giant kelp was at maximum biomass relative to when it was absent (0.08 vs. 0.55 kg C/m^2^/y).

Interestingly, total macroalgal NPP was highly variable at low giant kelp biomass (< 0.2 kg dry/m^2^) because of six observations under annual or quarterly disturbance with NPP values as high as those measured in control plots containing 5–100 times as much giant kelp biomass (shaded box in Figure 6a). Such remarkable observations were driven by very high understory productivity (shaded box in Figure 6b; 1.6–2.4 kg C/m^2^/y, the 95th–100th percentile of the data) that was much greater than the highest understory NPP observed in the absence of experimental disturbance (0.9 kg C/m^2^/y). Notably, these data all came from medium‐ and high‐quality sites with very low herbivore density (Mohawk, Isla Vista and Arroyo Quemado; Figure 1) during the later years of the experiment (5–10 years after start).

## DISCUSSION

Our results demonstrate that habitat quality can mediate disturbance‐driven changes to the rate and form of primary production. Disturbances commonly cause disproportionate losses from particular vegetation layers, but variation in resources, herbivory and other aspects of habitat quality may alter the effects of disturbance by changing both the amount of vegetation removed by disturbance and the potential for its lost productivity to be compensated by other vegetation layers (Halpern & Lutz, 2013; Roberts, 2004; Royo & Carson, 2006). By experimentally intensifying canopy disturbance along a gradient in grazing and substrate suitability, we discovered that the impacts of disturbance on kelp forest NPP increased with macroalgal habitat quality. Disturbance suppressed NPP by giant kelp and the total macroalgal community, but its effects were strongest in high‐quality habitats that supported dense kelp canopies that are more susceptible to disturbance. By reducing seafloor shading via canopy removal, disturbance enhanced the productivity of a diverse understory assemblage, and this effect magnified with increasing habitat quality. More generally, these findings contribute to a predictive understanding of how changing disturbance regimes and environmental conditions interact to alter NPP dynamics in systems with vertically stratified vegetation layers, including those dominated by long‐lived species that are difficult to study over multiple disturbance cycles, such as terrestrial forests.

### Effects of canopy disturbance on ecosystem productivity

By their sheer size and superior access to resources, canopy‐forming species are often highly productive and exert strong control over ecosystem productivity (Gower et al., 2001; Misson et al., 2007; Nilsson & Wardle, 2005; Tait & Schiel, 2018; Wiesner et al., 2019). By extension, disturbances that disproportionately damage the canopy, such as severe winds, fire, ice storms and large waves (Dayton et al., 1992; Reich et al., 2001; Roberts, 2004), should have direct negative effects on ecosystem productivity. Indeed, total macroalgal NPP closely tracked giant kelp biomass and its removal by disturbance greatly reduced the overall productivity of the kelp forest ecosystem. Our results echo those from rocky intertidal (Tait & Schiel, 2018) and temperate savannah (Reich et al., 2001) ecosystems, where frequent disturbances suppress total NPP by disproportionately reducing canopy species relative to the understory.

Because overlying canopies strongly influence resources available to lower vegetation layers (Hart & Chen, 2006; House et al., 2003), disturbances to canopy vegetation can also have indirect effects on ecosystem productivity by mediating canopy–understory competition. In our study, dense giant kelp canopies limited understory productivity by reducing light at the seafloor. This finding bolsters the general conclusion that understory productivity is light limited in many terrestrial and aquatic environments (Binzer et al., 2006; Feltrin et al., 2016; Hesketh & Baker, 1967). In coastal ecosystems throughout the world, kelp canopy shading suppresses understory macroalgal abundance (Clark et al., 2004; Santelices & Ojeda, 1984; Wernberg et al., 2005), but our uniquely comprehensive measurements are among the first to reveal the full effects of kelp canopies on macroalgal productivity, as they characterise NPP responses by entire macroalgal assemblages that incorporate spatial, seasonal and interannual variability. Long‐term NPP time series such as ours should help resolve significant uncertainties around the contributions of macroalgal productivity to coastal carbon sequestration and the ways that intensifying disturbance regimes will change the rate, form and fate of macroalgal production (Filbee‐Dexter & Wernberg, 2020; Krause‐Jensen & Duarte, 2016).

### Habitat quality mediates disturbance effects on understory productivity

Disturbance to giant kelp was especially effective in stimulating understory NPP in quarterly disturbances, which caused near continual absence of giant kelp, and at high‐quality sites, where sea urchin grazing and sand cover were low. In less favourable environments, understory NPP changed little in response to disturbance, even when giant kelp was significantly reduced, suggesting that herbivore density or the availability of hard substrate constrained understory responses. Similar patterns have been found in deciduous forests where overbrowsing limits understory shrub growth despite canopy disturbances that improve light (Royo & Carson, 2006), and in savannahs where soils and rainfall mediate the effects of canopy shading on herbaceous productivity (Frost et al., 1997; Hughes et al., 2006). Hence, although aspects of habitat quality differ among systems, there may be general support for the conclusion that habitat quality mediates the response of understory productivity to canopy loss.

### Productivity compensation among vegetation layers

Our results indicate that understory algae compensate to some extent for giant kelp canopy losses, and the degree of compensation is mediated by habitat quality. In poor‐quality habitat, productivity was low regardless of the experimental treatment, and thus, the effects of intensified disturbance on giant kelp NPP, understory NPP and their combined total were negligible. In disturbed plots in higher‐quality habitat, on the other hand, increases in understory NPP over time partly counteracted decreases in giant kelp NPP over time, resulting in no detectable temporal change in total macroalgal NPP relative to controls. Averaged across all years, experimental disturbance substantially reduced total macroalgal NPP at medium‐ and high‐quality sites, but such decreases would have been more severe had the understory not partly compensated for diminished giant kelp NPP.

After 5–10 years of giant kelp removal, understory assemblages at sites with low herbivore density periodically achieved very high productivity that boosted total macroalgal NPP to values comparable with controls. This suggests that while frequently disturbed kelp forests will on average be less productive than relatively undisturbed ones, understory assemblages occasionally can fully compensate for reductions in NPP arising from sustained canopy losses when conditions are ideal. By extension, factors that reduce herbivore densities, such as predation (Shears & Babcock, 2003), disease (Feehan & Scheibling, 2014) and unfavourable oceanographic conditions (e.g. temperature; Okamoto et al., 2020), should help bolster overall macroalgal NPP.

### Changes in the form and fate of NPP: Cascading community and ecosystem effects

Our results suggest that understory productivity, while usually less than that of the canopy, enhances ecosystem productivity and dampens fluctuations following canopy disturbances where environmental conditions allow, such as at sites with low herbivore density and suitable substrate (Lloyd et al., 2008; Nilsson & Wardle, 2005; O’Connell et al., 2003; Wiesner et al., 2019). However, the functional roles of understory and canopy NPP are not interchangeable because they differ in form and fate (Cebrian & Lartigue, 2004; Pace & Lovett, 2013). Giant kelp and understory macroalgal production have different routes through grazing and detrital food‐web pathways (Koenigs et al., 2015; Yorke et al., 2019). Likewise, much of the productivity of giant kelp and some understory macroalgae is exported across ecosystem boundaries to fuel secondary production on sandy beaches (Dugan et al., 2003) and in submarine canyons (Harrold et al., 1998; Krumhansl & Scheibling, 2012). Moreover, changes in the abundance of giant kelp and understory macroalgae affect a diverse community of reef fishes and invertebrates through non‐trophic mechanisms, such as providing refugia and foraging habitat (Castorani et al., 2018; Holbrook et al., 1990), and the role of giant kelp in creating and modifying habitat is important to consider above and beyond its unique ability to enhance primary productivity (Miller et al., 2018). Thus, even when understory macroalgae fully compensate for declines in giant kelp productivity, they cannot substitute for giant kelp's foundational attributes that enhance the diversity and stability of the kelp forest community and adjacent ecosystems (Castorani et al., 2018; Dugan et al., 2003; Lamy et al., 2020). Likewise, across a variety of terrestrial, freshwater and marine environments, many canopy‐forming species play distinct roles in structuring communities and ecosystems beyond solely altering primary productivity (Moffett, 2013; Olafsson, 2016).

### The value of long‐term experiments for understanding productivity

Our study highlights the inherent value of kelp forests for studying how disturbance shapes spatial and temporal patterns of NPP. Kelp forests have complex vertical structure formed by macroalgae that grow rapidly, reproduce often and respond quickly to changing disturbance regimes. These traits make kelp forests more amenable to experimentation than terrestrial counterparts that are typically characterised by slow‐growing species with long generation times and lengthy disturbance return intervals. And yet, it took 5–10 years of repeated canopy disturbance for large NPP changes to develop in our study. Giant kelp's high fecundity and rapid growth make it highly resilient to disturbance (Castorani et al., 2015, 2017), and thus, it is not surprising that it took several years for substantial decreases in canopy NPP to appear. Delayed, multiyear responses in understory NPP may be explained by the fact that the understory in our disturbance plots was dominated by slower‐growing perennial species (e.g. *Pterygophora californica*, *Stephanocystis osmundacea* and *Corallina officinalis*) that live 4–15 years or more (Dayton et al., 1984, 1992; Hymanson et al., 1990; Stewart, 1989).

Our results attest to the value of long‐term experimentation for understanding ecosystem responses to disturbance and environmental change. Had we carried out our study for less than 3 years – the duration of the vast majority of field experiments (Hobbie et al., 2003; Tilman, 1989) – we would have failed to quantify the magnitude of changes to understory and canopy productivity. Even for trends apparent after 3–4 years, we would have had low confidence that such patterns were not caused by natural variability or transient dynamics (Knapp et al., 2012; Tilman, 1989). In fact, although we had limited statistical power to identify saturating trends, our results suggest that giant kelp NPP would have continued to decline and understory NPP would have continued to rise beyond the 10‐year horizon of our study.

Manipulating forest canopy loss requires trade‐offs in the size and replication of experimental plots, the frequency and duration of experimentation and the complexity of physical and biological measurements. Our study had limited spatial replication (particularly at low habitat quality), but nevertheless improves understanding of how disturbance and habitat quality structure long‐term productivity dynamics. Spatial variation in habitat quality is rarely accounted for in estimates of macroalgal NPP, but is critically important when generalising within and among ecosystems (Krause‐Jensen & Duarte, 2016). Likewise, while our disturbance plots were relatively large, they could not perfectly emulate widespread canopy loss and may have been partly influenced by the surrounding unmanipulated kelp forest. Neighbouring giant kelp may have reduced current velocities in disturbance plots (Gaylord et al., 2007), but probably had little effects on nutrient delivery (Fram et al., 2008), wave attenuation (Elwany et al., 1995) or light availability (because of our sampling design); canopy removal plots were also unlikely to have attracted mobile fish and invertebrate consumers from the surrounding kelp forest (Castorani et al., 2018). Nevertheless, future studies may help contextualise our experimental findings with complementary approaches, such as comparing productivity across regions subject to different canopy disturbance regimes.

### Conclusions and future directions

Using kelp forests as a model ecosystem, our decade‐long experiment overcame several prior limitations to reveal that environmental gradients can mediate disturbance‐driven changes to canopy and understory productivity. Additional long‐term experiments are needed to further resolve how accelerating changes to both environmental conditions and disturbance regimes will alter primary productivity, and the extent to which management actions can moderate them (Dale et al., 2001; Gaiser et al., 2020; Harris et al., 2018). Future long‐term studies should embrace spatial variation to clarify the potential for understory vegetation to buffer losses in canopy productivity and determine the timescales of such compensation (House et al., 2003).

## AUTHORSHIP

DCR designed the experiment. SLH and DCR led the data collection and compilation. MCNC designed and performed the analyses and wrote the manuscript. DCR, RJM and SLH contributed to interpreting the results and revising the manuscript. All authors gave final approval for publication. The authors declare that they have no competing interests.

## Supporting information

Supplementary Material

## Data Availability

All data and metadata used in this study are publicly available through the Environmental Data Initiative (https://environmentaldatainitiative.org/). Macroalgal biomass data are available from Reed and Miller (2021b). Sand cover data are available from Reed and Miller (2021c). Sea urchin density data are available from Reed and Miller (2021d). Light data are available from Reed et al., (2021). Taxon‐specific NPP data are available from Harrer et al., (2021). The data DOIs are cited in the Reference list.
