# Supplementary material for: Disturbance structures canopy and understory productivity along an environmental gradient
Source: Ecol Lett. 2021 Aug 2;24(10):2192–206. doi: 10.1111/ele.13849 (PMC8518717; doi:10.1111/ele.13849)
Supplement: Supplementary file 1 — Supplementary Material [file ELE-24-2192-s001.pdf]

Supporting Information

Disturbance structures canopy and understory productivity along an environmental gradient

Max C. N. Castorani, Shannon L. Harrer, Robert J. Miller, and Daniel C. Reed

Appendix S1: Detailed methods of productivity estimation.

Overview. We estimated the daily net primary productivity (NPP) of macroalgae in each plot by combining time series data on *in situ* taxon-specific biomass and hourly irradiance with taxon-specific relationships between irradiance and photosynthesis, accounting for respiration. For juvenile giant kelp *Macrocystis pyrifera* (< 1 m tall) and understory macroalgae (56 taxa), we estimated NPP using field measurements of irradiance and biomass (derived from abundance) and laboratory estimates of taxon-specific photosynthetic parameters. For adult giant kelp (> 1 m tall), we converted field measurements of frond density to biomass and NPP using month-specific linear relationships developed for our study region (Rassweiler *et al.* 2018).

Macroalgal biomass sampling. Divers measured macroalgal biomass within a permanent 40 m × 2 m transect located in the center of each plot once per season as defined by the typical solar solstice and equinox dates: winter (Dec. 21–Mar. 19), spring (Mar. 20–June 20), summer (June 21–Sep. 21), and autumn (Sep. 22–Dec. 20). Macroalgae were surveyed in disturbance treatment plots prior to each experimental removal of giant kelp.

Different nondestructive methods were used to quantify macroalgal abundance depending on species size and morphology (detailed methods in Reed & Miller 2021a, b, c). Within each transect, divers counted the number and measured the size of all giant kelp and larger understory kelps and fucoids: *Laminaria farlowii*, *Pterygophora californica*, *Ecklonia arborea*, *Egregia menziesii*, *Sargassum horneri*, *Sargassum muticum*, and *Stephanocystis osmundacea*. The

abundances of smaller kelps were subsampled in six permanent 1 m^2 quadrats spaced uniformly along each transect. Abundances of small or clonal macroalgae that are difficult to distinguish as individuals were measured as percent cover using a grid of 80 points spaced uniformly within a 1 m wide band spanning each transect. These taxa include the following crustose algae, low lying turfs, and understory foliose algae: *Bossiella orbigniana*, *Callophyllis flabellulata*, *Chondracanthus corymbiferus*, *Corallina chilensis*, large *Stephanocystis osmundacea* (diameter > 10 cm), *Desmarestia ligulata*, *Laurencia spectabalis*, *Polyneura latissima*, *Rhodymenia californica*, *Dictyota* spp., family Ectocarpaceae, *Polysiphonia* spp., *Pterosiphonia* spp., *Halymenia* spp., and crustose coralline algae consisting primarily of *Pseudolithophyllum neofarlowii*.

We converted size-specific abundance and percent cover measurements of juvenile giant kelp and understory macroalgae to biomass (g dry m^{-2}) using taxon-specific relationships derived from data and samples collection from 11 reefs in the region (Nelson *et al.* 2021). Relationships for the invasive fucoid *Sargassum hornerii* were derived using data and tissue samples from Catalina Island, California, USA. We converted adult giant kelp frond density to dry mass using month-specific allometric relationships developed for the region (Rassweiler *et al.* 2018).

Irradiance sampling. We measured light once or twice per minute using a photosynthetically active radiation (PAR) logger-sensor placed 30 cm above the seafloor within each transect (Reed *et al.* 2021). Sensors were retrieved for data download and servicing every 6–12 weeks and simultaneously replaced with newly serviced sensors. During deployment, biological fouling (primarily by benthic diatoms) on the sensors occurred to varying degrees. To account for inaccuracies due to biofouling, we cleaned sensors *in situ* 20 minutes before retrieval and calculated attenuation by biofouling as

$$a = -\ln(I_{dirty}/I_{clean}),$$

where I_{dirty} is the mean irradiance sampled once per minute for 20 minutes prior to the sensor being cleaned on retrieval day and I_{clean} is the mean irradiance sampled once per minute for 20 minutes immediately after cleaning. The effects of biofouling on irradiance were assessed by comparing mean I_{dirty} and mean I_{clean} using a student's t-test ($\alpha = 0.05$). Irradiance values from significantly fouled sensors were corrected on each day of the deployment as

$$I_{corrected} = I_{measured} \cdot e^{\frac{a}{\sum d}} \cdot t,$$

where a represents attenuation due to fouling as described above, $\sum d$ represents the total number of days since deployment (over which we assume the fouling accumulated), and t represents the number of days that have passed since deployment for the set of values being corrected (Harrer *et al.* 2013).

Beginning in 2016, spherical PAR sensors (MDS-MkV/L, Alec Electronics, Kobe, Japan) were replaced with planar PAR sensors (DEFI-L, Alec Electronics, Kobe, Japan). Data collected during simultaneous deployments of paired spherical and planar sensors were used to develop algorithms to convert all values recorded from planar sensors to values representative of spherical sensors (detailed methods in Reed *et al.* 2021).

Surface irradiance was measured from ~30 to 100 cm above the sea surface on a moored vertical spar buoy at three reef sites (Arroyo Quemado, Carpinteria and Mohawk). Time series measurements demonstrated that sea surface irradiance values were extremely similar among sites. Due to these similarities and the fact that surface sensors proved difficult to maintain due to sensor damage and loss caused by storms and boat traffic, in 2016 we began using a single surface sensor deployed on an unobstructed rooftop at the nearby University of California, Santa

Barbara. Surface sensors were calibrated for readings in air by the manufacturer and not adjusted for biofouling because it did not occur.

Sensor malfunction and availability caused data gaps in seafloor irradiance in some plots (Reed *et al.* 2021). To account for these missing data in NPP calculations, we estimated seafloor irradiance by calculating water column attenuation from surface irradiance and seafloor irradiance in other plots during the period of missing data. All estimates of attenuation were constrained to be positive and below infinity by converting to very low values (0.01 μmol m^{-2} s^{-1}) in cases where surface irradiance was positive, but seafloor irradiance was measured to be 0 (i.e., below the limit of detection). In situations where surface irradiance was low (< 100 μmol m^{-2} s^{-1}), we constrained attenuation to be positive by converting seafloor irradiance to 6% of measured surface irradiance.

Because attenuation varies in space and time due to variation in kelp biomass and other factors, we determined the best estimate of attenuation for filling data gaps in seafloor irradiance. Hourly seafloor irradiance for a given plot was estimated using the mean attenuation (across all years) for that hour and day of the year. If data gaps remained in a quarterly disturbance plot, we estimated hourly seafloor irradiance using attenuation data collected from the adjacent annual disturbance plot at that site. Likewise, if data gaps remained in an annual disturbance plot, we estimated hourly seafloor irradiance using attenuation data collected from the adjacent quarterly disturbance plot at that site. If data gaps still remained in a given plot, we estimated hourly seafloor irradiance using the mean attenuation for that hour and day of the year averaged over all years for that plot.

We used seafloor irradiance to calculate NPP of all understory taxa, except for the kelp *Egregia menziesii* and reproductive fronds of the fucoid *Stephanocystis osmundaceae*, which

grow to midwater depths. For these two species, we coupled surface irradiance measurements with estimates of water-column attenuation to approximate midwater irradiance (i.e., at one-half of the sampling depth). For low values of surface irradiance (< 100 μmol m^{-2} s^{-1}), we assumed midwater irradiance was 20% of surface irradiance.

Physiological measurements. Following Miller *et al.* (2012), we measured photosynthesis versus irradiance and respiration for the 22 most commonly observed understory macroalgal taxa (Harrer *et al.* 2020), which accounted for 97% of total understory biomass in our study. We also measured photosynthesis versus irradiance for the reproductive fronds of the fucoid *Stephanocystis osmundaceae*, which can exhibit seasonally high biomass. We used these estimates of net photosynthesis at saturating irradiance ($P_{\max}$), net photosynthesis at non-saturating irradiance (α), and respiration in our calculations of hourly NPP. We estimated values for the photosynthetic parameters of less common taxa (accounting for the remaining 3% of understory biomass) using those based on measurements of common morphologically-similar species (Harrer *et al.* 2021).

Estimation of NPP from biomass and irradiance. For juvenile giant kelp and each understory taxon *i* in each plot, we calculated daily NPP (g C m^{-2} d^{-1}) as the sum of gross production and respiration over all daylight hours and respiration over all hours of darkness at each sampling location for each day of the year after Miller *et al.* (2012), which followed a modified version of the equation of Jassby and Platt (1976):

$$\mathrm{NPP}_i = \sum_h P_{max} \cdot \tanh\left(\frac{\alpha_i E_h}{P_{max}}\right) \cdot b_i \; - \sum_h R \cdot b_i \;,$$

where P_{max} is net photosynthesis at saturating irradiance (mg C h^{-1} [g dry mass]$^{-1}$), α_i is net photosynthesis at non-saturating irradiance (mg C h^{-1} [g dry mass]$^{-1}$ [μmol m^{-2} s^{-1}]$^{-1}$), E_h is mean seafloor irradiance (μmol m^{-2} s^{-1}) over the course of an hour (*h*), *R* is respiration in the dark (mg

C h^{-1} [g dry mass]$^{-1}$), and b_i is the daily estimate of standing dry biomass (g m^{-2}) of an individual taxon *i*. We estimated b_i using linear interpolations of biomass from one sampling date to the next.

We estimated daily NPP of adult giant kelp by multiplying the interpolated value of its biomass for that day by the slope of the relationship between biomass and mean daily NPP developed for each month of the year by Rassweiler et al. (2018). For days during sampling periods that began immediately following experimental removal of all giant kelp, the interpolated value of giant kelp biomass was calculated using an initial value of zero. Following Roxburg et al. (2005), for all taxa we constrained daily NPP to zero when daily respiration exceeded daily production. We summed taxon-specific estimates of daily NPP from early spring of one calendar year (after initiating experiments in midwinter) through late winter of the following calendar year to produce annual NPP values (g C m^{-2} y^{-1}) for each year following the start of the experiment.

References

Harrer, S.L., Reed, D.C., Holbrook, S.J. & Miller, R.J. (2013). Patterns and controls of the dynamics of net primary production by understory macroalgal assemblages in giant kelp forests. *J. Phycol.*, 49, 248–257.

Harrer, S.L., Reed, D.C. & Miller, R.J. (2020). SBC LTER: Reef: Macroalgal photosynthetic parameters ver 9. *Environ. Data Initiat.*, https://doi.org/10.6073/pasta/3c99428af96eae608398d111f982782a.

Harrer, S.L., Reed, D.C., Miller, R.J. & Holbrook, S.J. (2021). SBC LTER: Reef: Long-term experiment: Taxon-specific seasonal net primary production (NPP) for macroalgae ver 14. *Environ. Data Initiat.*, https://doi.org/10.6073/pasta/0219f79d9e6d9485a892a669a8ce23b8.

Jassby, A.D. & Platt, T. (1976). Mathematical formulation of the relationship between photosynthesis and light for phytoplankton. *Limnol. Oceanogr.*, 21, 540–547.

Miller, R.J., Harrer, S. & Reed, D.C. (2012). Addition of species abundance and performance predicts community primary production of macroalgae. *Oecologia*, 168, 797–806.

Nelson, C.J., Reed, D.C., Harrer, S.L. & Miller, R.J. (2021). SBC LTER: Reef: Coefficients for estimating biomass from body size or percent cover for kelp forest species ver 3. *Environ. Data Initiat.*, https://doi.org/10.6073/pasta/0fe9233dabe35df5d61fb3b07f8fb51e.

Rassweiler, A., Reed, D.C., Harrer, S.L. & Nelson, J.C. (2018). Improved estimates of net primary production, growth and standing crop of *Macrocystis pyrifera* in Southern California. *Ecology*, 99, 2440–2440.

Reed, D.C., Harrer, S.L. & Miller, R.J. (2021). SBC LTER: Kelp Removal Experiment: Hourly photon irradiance at the surface and seafloor ver 19. *Environ. Data Initiat.*, https://doi.org/10.6073/pasta/803abbcd7fb33bbfa9eff08521a397e8.

Reed, D.C. & Miller, R.J. (2021a). SBC LTER: Kelp Forest Community Dynamics: Cover of sessile organisms, Uniform Point Contact ver 29. *Environ. Data Initiat.*, https://doi.org/10.6073/pasta/adab61f8b0da4504a389c63fee17f866.

Reed, D.C. & Miller, R.J. (2021b). SBC LTER: Reef: Long-term experiment: Biomass of kelp forest species, ongoing since 2008 ver 7. *Environ. Data Initiat.*, https://doi.org/10.6073/pasta/0e4d59bfe757781ed51661267865af5c.

Reed, D.C. & Miller, R.J. (2021c). SBC LTER: Reef: Long-term experiment: Kelp removal: Invertebrate and algal density ver 18. *Environ. Data Initiat.*, https://doi.org/10.6073/pasta/b8c0201a94fd6cb91ed4c1ce574d89f2.

Roxburgh, S.H., Berry, S.L., Buckley, T.N., Barnes, B. & Roderick, M.L. (2005). What is NPP? Inconsistent accounting of respiratory fluxes in the definition of net primary production. *Funct. Ecol.*, 19, 378–382.

Appendix S2: Figure S2. Giant kelp biomass experimentally removed in annual (top) and quarterly (bottom) disturbance plots at sites of varying habitat quality. Polygons are violin plots showing the estimated kernel probability density of the data. Black boxes are boxplots where the width of the box shows the interquartile range (IQR; 25th to 75th percentile) and whiskers extend above and below the IQR by 1.5 × IQR. Black points show outliers beyond 1.5 × IQR. White points show the median of the data. Note that the top panel shows the amount of giant kelp biomass removed per year, whereas the bottom panel shows the amount of giant kelp removed per quarter.

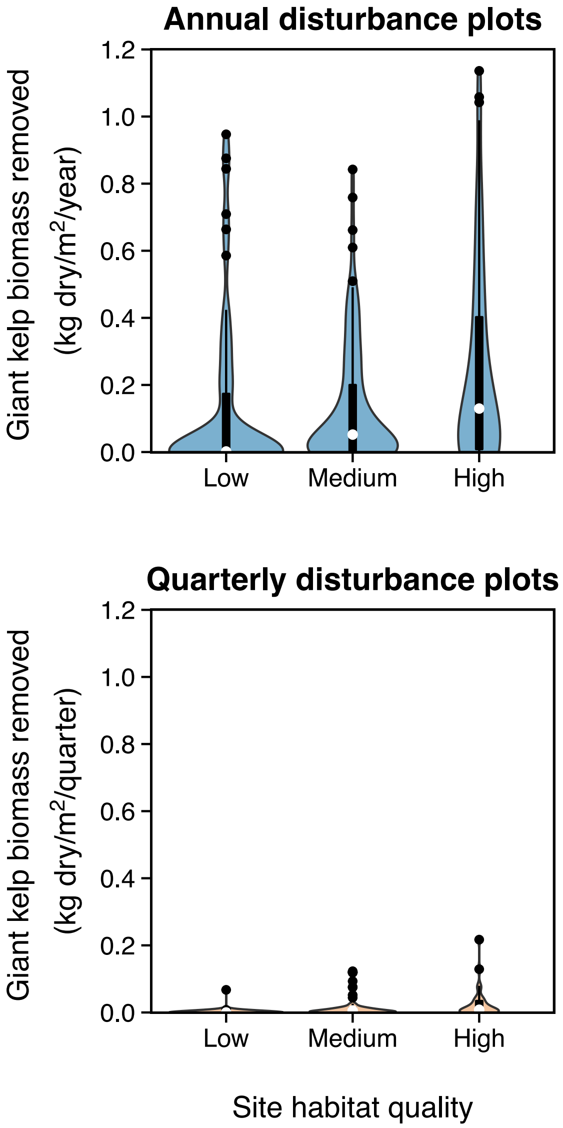

Appendix S3: Figure S3. Sea urchin density and sand cover had negative effects on total macroalgal biomass. Data represent ten years (2008–2018) of seasonal observations from unmanipulated control plots in five kelp forest sites near Santa Barbara, California, USA. Line and shading show estimated relationship and 95% confidence interval, respectively.

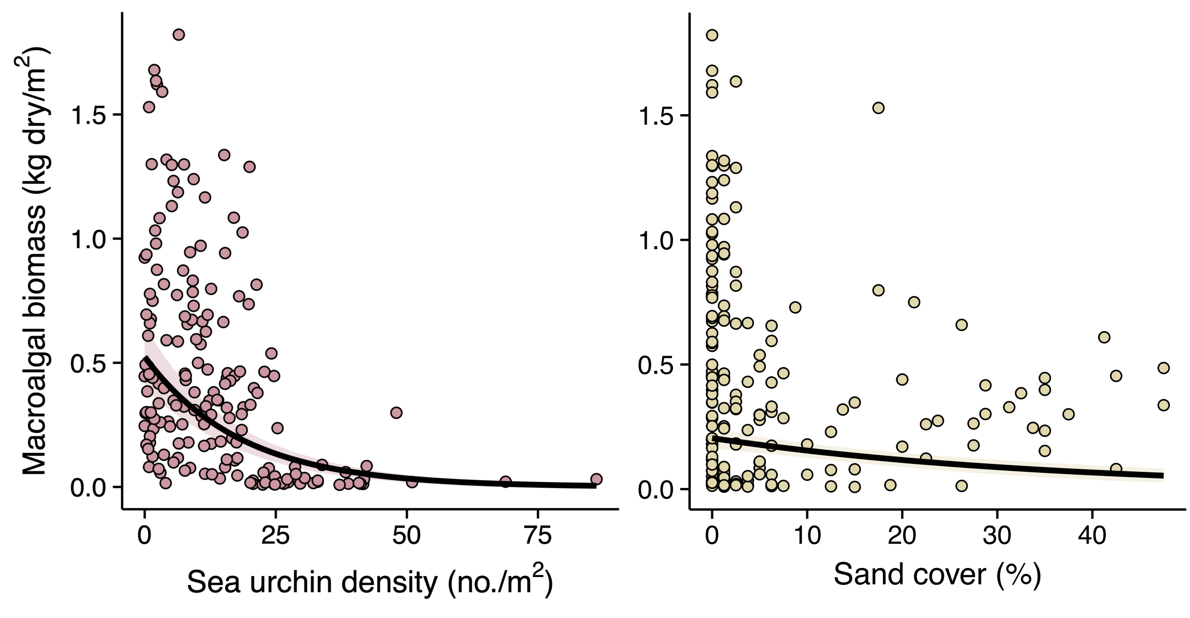

Appendix S4: Table S4. Results of analysis of bottom light within unmanipulated control plots.

Source of variation	df	χ^2	*P*
Giant kelp biomass	1	76.2	**< 0.001**
Giant kelp biomass × Season	3	17.4	**0.001**
Season	3	114.0	**< 0.001**
Site	4	28.1	**< 0.001**
Year	1	7.1	**0.013**
Residual	225		

Notes: Bold face indicates P < 0.05.

Appendix S5: Figure S5. The amount of light reaching the kelp forest seafloor declined exponentially with increasing giant kelp biomass across all seasons ($P < 0.001$). This effect was strongest in spring and summer, weakest in autumn, and intermediate in winter. Data represent seasonal measurements in control plots at all five sites averaged over 15-day windows centered on point estimates of giant kelp biomass. Lines and shading show estimated relationships and 95% confidence intervals, respectively.

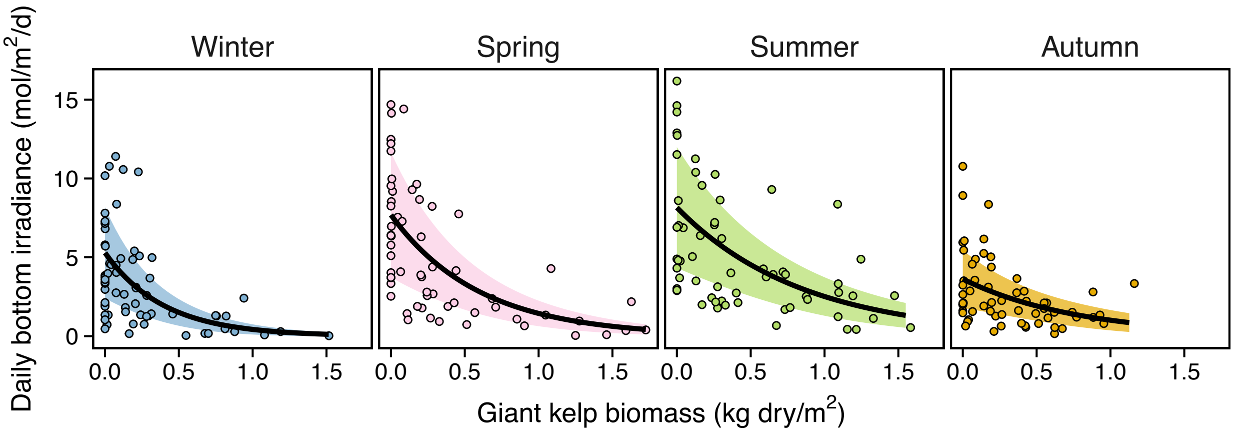

Appendix S6: Table S6. Results of analysis of annual net primary productivity (NPP) of giant kelp, understory macroalgae, and their combined total in disturbance plots relative to paired control plots as a function of disturbance treatment (annual or quarterly), habitat quality (low, medium, or high), and years since the start of the experiment.

	Giant kelp NPP			Understory NPP			Total macroalgal NPP		
Source of variation	df	χ^2	*P*	df	χ^2	*P*	df	χ^2	*P*
Treatment	1	17.9	**< 0.001**	1	0.7	0.5	1	17	**< 0.001**
Habitat quality	2	111.1	**< 0.001**	2	104.8	**< 0.001**	2	63.4	**< 0.001**
Years since start	1	19.1	**< 0.001**	1	39.5	**< 0.001**	1	3.9	0.08
Treatment × Habitat quality	2	2.0	0.4	2	11.3	**0.006**	2	0.4	0.8
Treatment × Years since start	1	0.1	0.8	1	10	**0.003**	1	1.8	0.2
Habitat quality × Years since start	2	1.4	0.6	2	16	**0.001**	2	1.6	0.5
Treatment × Habitat quality × Years since start	2	2.9	0.3	2	13.3	**0.003**	2	5.3	0.1
Residual	56			58			56		

Notes: Bold face indicates P < 0.05.

Appendix S7: Figure S7. Patterns of annual net primary productivity (NPP) by (A) giant kelp, (B) understory macroalgae, and (C) their combined total as a function of experimental treatment and site habitat quality. Left panels shown means across all years ± bootstrap 95% confidence intervals. Right panels show means over time since the start of the experiment.

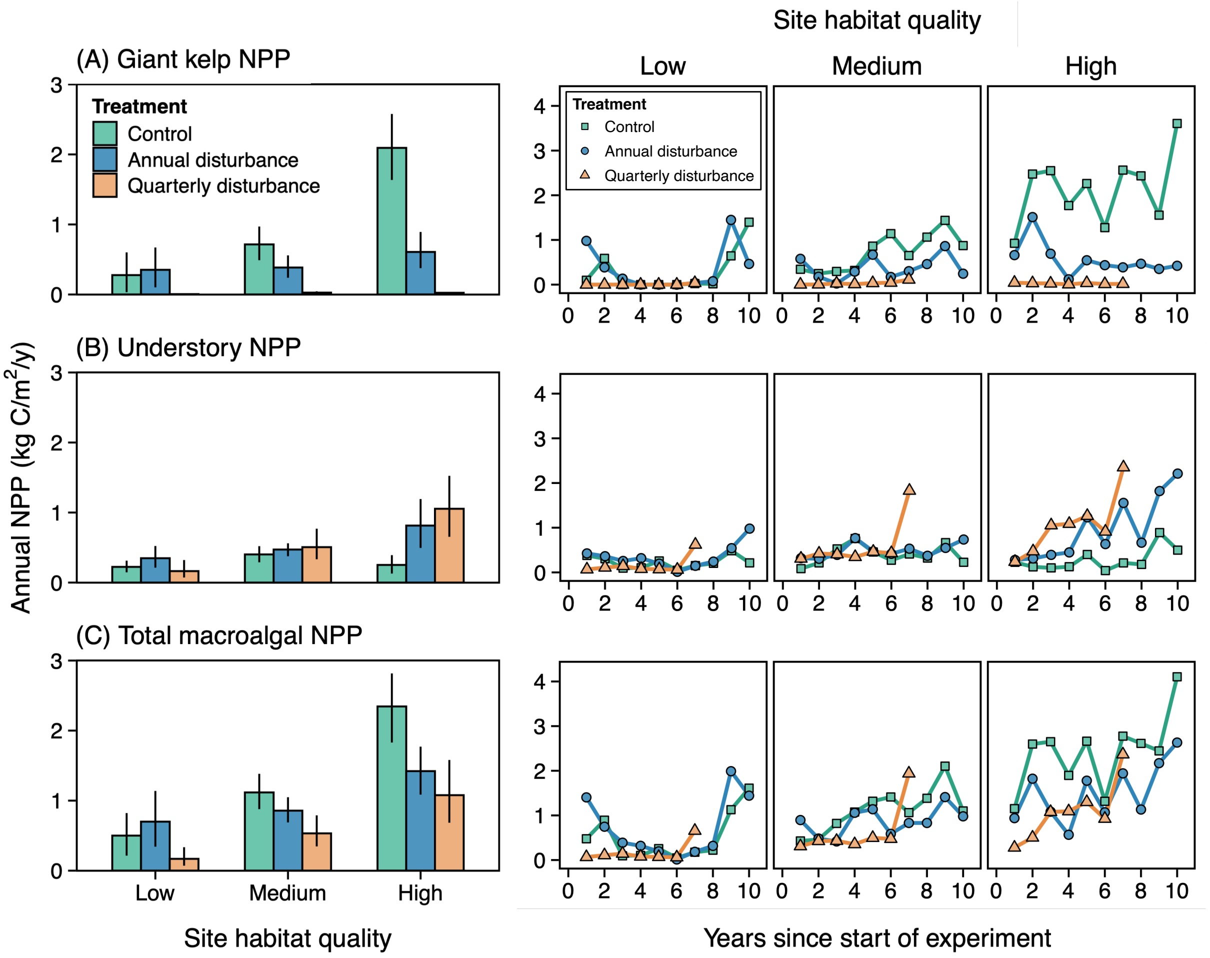

Appendix S8: Figure S8. Annual total macroalgal net primary productivity (NPP) in disturbance plots relative to control plots as a function of disturbance regime, habitat quality, and years since the start of the experiment. Horizontal dashed line indicates equal annual NPP in paired control and disturbance plots.

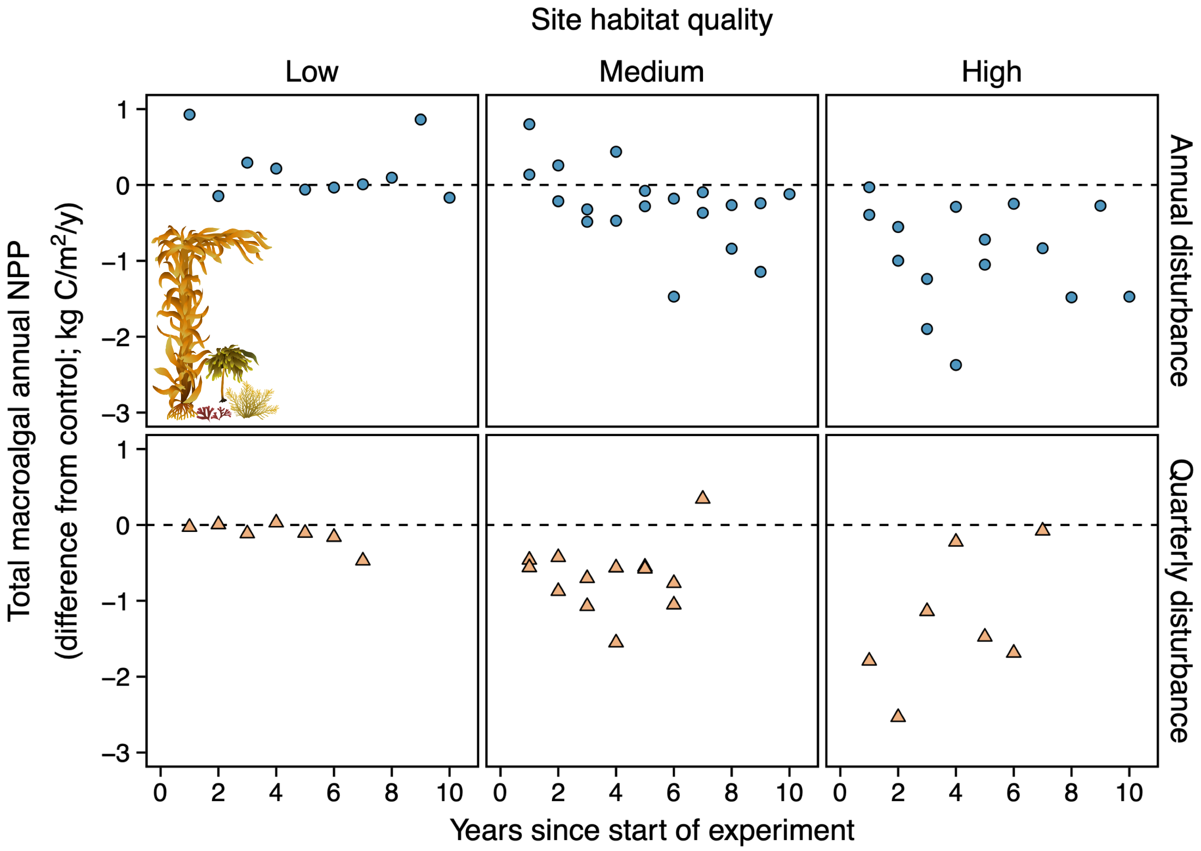

Appendix S9: Table S9. Results of analysis of annual understory and total macroalgal net primary production (NPP) as a function of giant kelp biomass and covariates.

	Understory NPP			Total macroalgal NPP		
Source of variation	df	χ^2	*P*	df	χ^2	*P*
Giant kelp biomass	1	25.2	**< 0.001**	1	224.0	**< 0.001**
Sea urchin density	1	10.9	**0.002**	1	1.3	0.3
Proportional sand cover	1	0.5	0.6	1	0.4	0.6
Year	1	5.0	**0.042**	1	7.0	**0.013**
Residual	107			101		

Notes: Bold face indicates P < 0.05.
